# Understanding undernutrition

**Published:** 2013

For children to have adequate micronutrient intake, including vitamin A, all of the following should be in place.

1. **Nutrient-rich foods must be available to the family**. Sufficient nutrient-rich foods must be available where the family can buy them, and must be affordable.
2. **Children must consume enough nutrient-rich foods**. Children must be given enough nutrient-rich food to eat. Food must be prepared in such a way that the nutrients are preserved and easily absorbed. For most foods, this means not overcooking them. For good vitamin A absorption, there must also be enough fat in the diet.
3. **Children must be free from disease and infection**. Measles, intestinal worms, diarrhoea and other infections all reduce the absorption – and increase the loss – of many micronutrients, including vitamin A.

## What can go wrong?

### 1 Nutrient-rich foods are not available or affordable

If this is the case, children will not get adequate amounts of micronutrients, including vitamin A, even if parents prepare the foods correctly and the children are free of disease. There may be several underlying causes:

- the foods may be seasonal
- markets may be far or transport is a problem
- the family may not own land so cannot grow their own crops
- some foods, such as meat and other animal products, are not affordable for poorer families.

### 2 Nutrient-rich foods are not given to children, are not given in the correct way, and/or children do not want to eat these foods

This can be due to a combination of factors, including social and/or cultural norms and beliefs, poverty and lack of knowledge. Here are some examples:

- mothers may not know which foods are healthy for their young children
- mothers may stop breastfeeding early and give complementary foods low in nutrients
- nutrient-rich foods may be overcooked
- dark green leafy vegetables (high in vitamin A) are not cooked in such a way that children will eat them
- there may be local customs about what to feed children who are sick, e.g. porridge instead of vegetables
- local customs may stop mothers giving children certain foods; e.g. a belief that eggs cause children to become thieves
- if there is not enough food for the family, the more nutritious food may be given to the head of the household.

### 3 Children suffer from disease and infection

Disease and infection can:

- reduce the body's ability to absorb nutrients from food
- increase the body's demand for nutrients
- increase the loss of nutrients from the body.

**Absorption** is reduced by:

- gastrointestinal tract infections, diarrhoea and intestinal worms
- chronic undernutrition itself.

**Demand** is increased by:

- repeated episodes of infection with fever
- measles infection. Vitamin A is needed to repair cells damaged in the skin, lungs, gut, mouth, conjunctiva and middle ear. In children with inadequate vitamin A intake, measles infection can very quickly deplete the body's vitamin A stores (in the liver), leading to blindness, hearing impairment and death.

**Loss** is increased by:

- diarrhoea: micronutrients are lost from the gut.
- measles: retinol is lost in the urine or from the gut.

**Figure:**
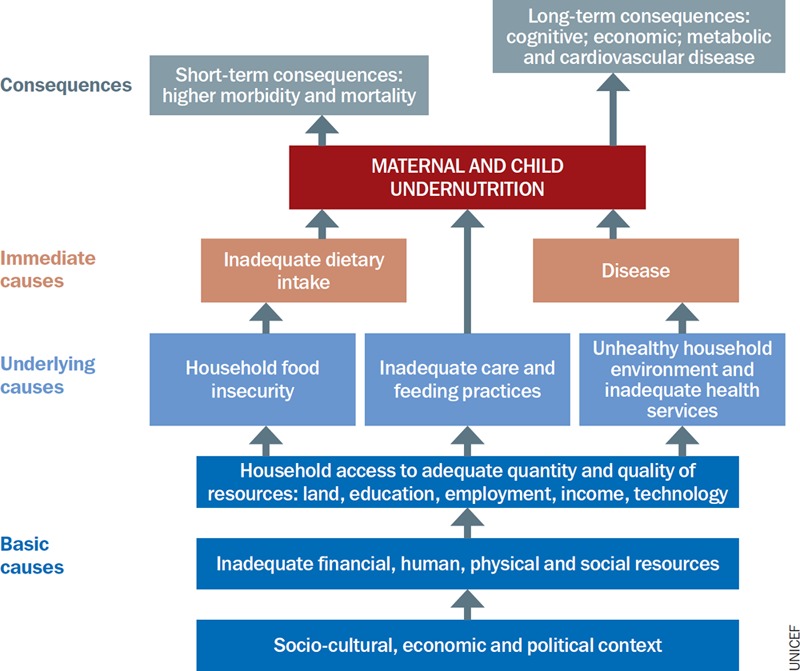
Figure 1. Conceptual framework of the determinants of child undernutrition^1^

## Underlying causes

There are a range of underlying causes of undernutrition, as shown in Figure 1:

- food insecurity at household level
- lack of knowledge about good health, hygiene and child feeding practices
- lack of sanitation and water, leading to an unhealthy household environment
- inadequate health services, including low coverage of measles immunisation programmes and a lack of primary care.

These causes are themselves the result of more basic causes, e.g. a lack of access to resources including income, education, land, and technology. The social, cultural, economic and political context people find themselves in also plays a role.

Although these causes are difficult to address, as eye care providers we can do much to address misconceptions and improve knowledge about nutrition among parents and in communities (see pages 72–73). We can promote good hygiene practices, measles immunisation and deworming and encourage parents to give their children a diet that is nutritious and rich in micronutrients, including vitamin A.
